# Prognostic and Clinicopathological Value of Ki-67 in Melanoma: A Meta-Analysis

**DOI:** 10.3389/fonc.2021.737760

**Published:** 2021-09-08

**Authors:** Qixin Liu, Ziheng Peng, Liangfang Shen, Lin Shen

**Affiliations:** ^1^Department of Oncology, Xiangya Hospital, Central South University, Changsha, China; ^2^Department of Gastroenterology, Xiangya Hospital, Central South University, Changsha, China

**Keywords:** melanoma, Ki-67, prognosis, clinicopathology, meta-analysis

## Abstract

**Background:**

The prognostic and clinicopathological value of Ki-67 in melanoma is controversial. The purpose of this meta-analysis was to determine the prognostic role of Ki-67 in melanoma patients.

**Materials and Methods:**

The PubMed, Cochrane Library, Web of Science, and Embase databases were searched systematically up to April 9, 2021. We calculated the pooled hazard ratios (HRs) and 95% confidence intervals (CIs) to determine the relationship between Ki-67 overexpression and survival outcomes. We also calculated the combined odds ratios (ORs) and 95% CIs to determine the relationship between Ki-67 expression levels and clinicopathologic parameters. All data were statistically analyzed by Stata 11.0.

**Results:**

A total of 10 studies involving 929 patients were included in our meta-analysis. The pooled HR showed that Ki-67 overexpression was connected with poor overall survival rates (HR=2.92, 95% CI=2.17-3.91, p<0.000). However, there was no correlation between Ki-67 overexpression and the PFS (HR=0.999, 95% CI =0.958-1.041, P =0.958; I2 = 21.80%, P =0.258) or RFS (HR=1.14, 95% CI = 0.42-3.11, P =0.993; I2 = 85.00%, P =0.01) rates. Ki-67 expression levels were associated with tumor thickness, but not sex, location, ulceration or vascular invasion.

**Conclusion:**

Ki-67 is a useful poor prognostic indicator for melanoma patients.

## 1 Introduction

Melanoma is a diffuse neuroendocrine tumor originating from the neural crest that mainly occurs in the skin and mucosa. The global number of melanoma cases increased from 232,000 in 2012 to 351,880 in 2015, and 62,000 patients died from melanoma in 2015. As the most common cutaneous malignant tumour, melanoma has the characteristics of high malignancy levels, increasing morbidity and mortality rates, and extremely high treatment costs (1). Ethnicity, sun exposure, alcohol consumption, vitamin D deficiency, obesity and exposure to chemicals such as oil and pesticides have all been cited as causes of melanoma (2). With the continuing rise in morbidity, melanoma has become a challenging public health problem worldwide, especially in New Zealand, Australia, Norway, Sweden, and the Netherlands (3). Moreover, melanoma is projected to become the second most common cancer in the United States by 2040 (4). The global incidence of melanoma has increased observably in recent years; however, some emerging therapies, such as immune checkpoint inhibitors, have led to a significant decline in melanoma patient mortality rates (5).

As a nuclear protein expressed in proliferating mammalian cells, Ki-67 controls gene expression by organizing heterochromatin spatially (6). Immunohistochemical (IHC) staining is usually used to detect Ki-67, and the expression level of Ki-67 is related to cell proliferation activity, disease progression and cancer recurrence (7). Some studies have shown that Ki-67 is one of the prognostic indices of multiple solid tumors, such as nasopharyngeal carcinoma (8), stage I non-small cell lung cancer (9), gastrointestinal stromal tumour (10), and gliomas (11), resected triple-negative breast cancer (12), colorectal cancer (13), hepatocellular carcinoma (14), and thyroid cancer (15). Previous studies have shown an association between Ki-67 expression and melanoma patient prognosis, but the results have been contradictory (16–25). Some studies show that high Ki-67 expression is an indicator of worse prognosis (16–22, 25), while other studies suggest that high Ki-67 expression predicts favorable prognosis (23, 24). Therefore, we conducted this meta-analysis to accurately determine the prognostic and clinicopathological significance of Ki-67 in melanoma patients to optimize treatment strategies.

## 2 Materials and Methods

This meta-analysis was conducted on the basis of the Preferred Reporting Items for Systematic Reviews and Meta-Analyses statement (26).

### 2.1 Search Strategy

The PubMed, Cochrane Library, Web of Science, and Embase databases were searched systematically up to April 9, 2021. Because the data in this study were extracted from previous studies, ethical approval and patient consent were not required. The search terms were as follows: (melanoma or malignant melanoma or melanocytoma) and (Ki67 or Ki-67 or MIB-1 or MIB1).

### 2.2 Inclusion and Exclusion Criteria

The inclusion criteria were as follows: (1) histopathology confirmed the diagnosis of melanoma; (2) the expression of Ki-67 in tissues was detected by immunohistochemistry (IHC); (3) at least one survival outcome was reported, such as the overall survival (OS), progression-free survival (PFS), or relapse-free survival (RFS) rate with hazard ratio (HR) and 95% confidence interval (CI); and (4) studies were published in English or Chinese.

The exclusion criteria were as follows: (1) reviews, letters, case reports, expertise public opinion and conference abstracts; (2) studies on tumor cell lines and animal models; (3) duplicate studies or duplicate data; and (4) studies that did not provide necessary and complete data.

### 2.3 Data Extraction and Quality Assessment

Two independent researchers read the eligible studies and extracted basic information independently; any differences were settled through repeated discussion. The following information was extracted: author, country or region, sample size, sex, age, study type, Ki-67%, AJCC stage, Clark level, follow-up and HRs and 95% CIs of OS, PFS, and RFS rates. Some HRs and 95% CIs could be obtained directly from the studies, while others were calculated from the survival curves. The quality of the selected articles was assessed using the Newcastle Ottawa Scale (NOS) criteria (27). NOS scores range from 0 to 9, and studies with a score of 6 are considered high-quality studies; otherwise, they are considered low-quality studies.

### 2.4 Statistical Analysis

The statistical analysis was conducted by Stata SE11.0. The HR and 95% CI were used to estimate the relationship between the Ki-67 value and survival outcomes, including the OS, PFS and RFS rates. ORs (odds ratios) and 95% CIs were used to evaluate the relationship between the Ki-67 value and the clinical characteristics of the melanoma patients, such as sex and location and tumor ulceration, thickness, and vascular invasion. We performed subgroup analyses, as shown in **Table 4**, by stratifying the combined data according to region (Europe and America *versus* Asian), patients (≥100 *versus* <100), median age (≥50 years *versus* <50 years), study type (prospective *versus* retrospective), Ki-67% (≥25% *versus* <25%), and follow-up time (>=48 months *versus* <48 months). Cochran’s Q statistic and *I* ^2^ statistic were used to quantify the heterogeneity among the studies. The random-effects model was applied when the heterogeneity was significant (*I* ^2^> 50%); otherwise, a fixed-effects model used. Begg’s test was performed to assess potential publication bias. A p-value <0.05 was considered statistically significant.

## 3 Results

### 3.1 Search Result and Research Characteristics

**Figure 1:**
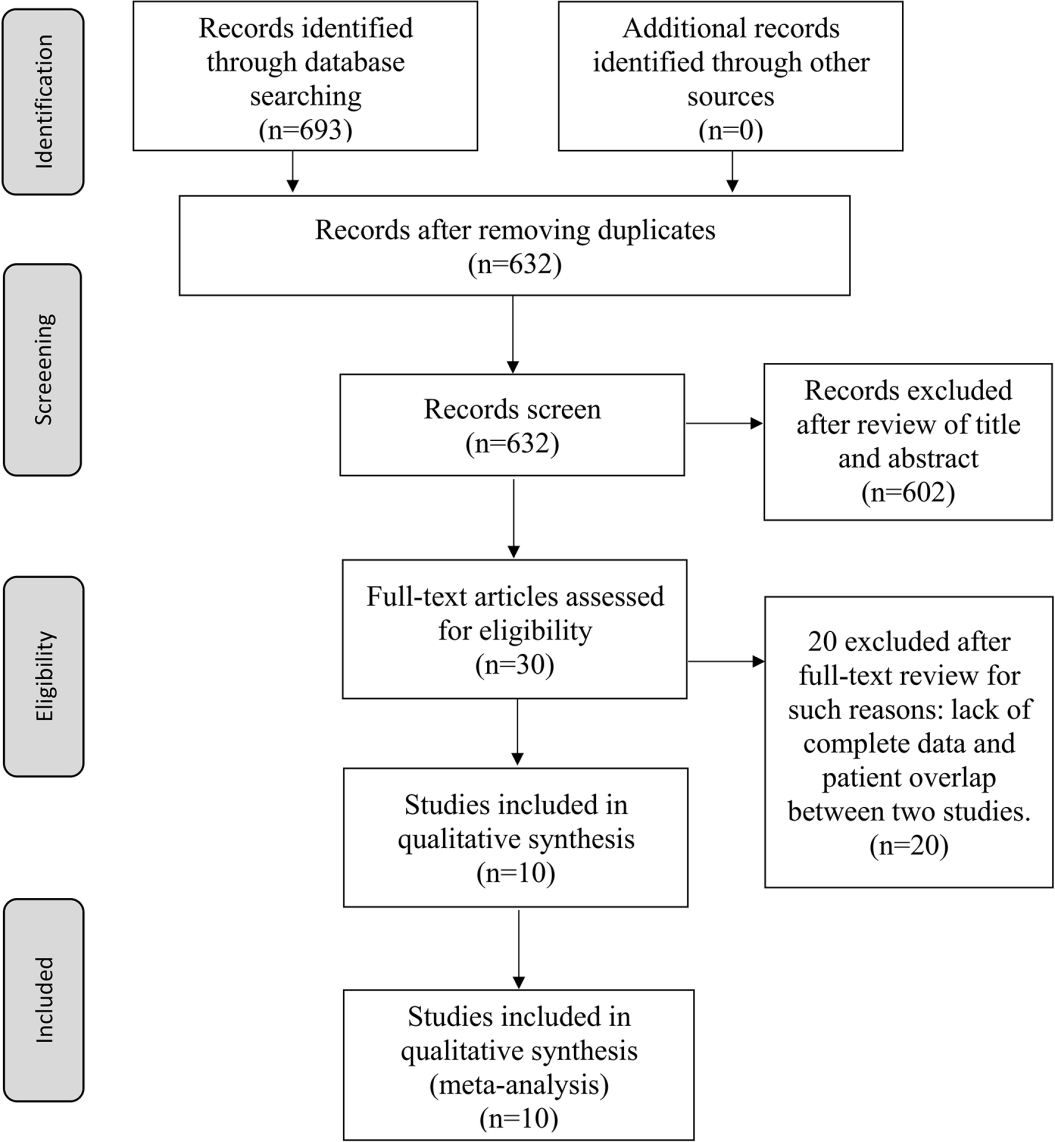
Flow diagram of reviewing and selecting studies.

The flow chart of the article retrieval process is presented in **Figure 1**. A total of 693 records were found through an initial search. After deleting 61 duplicate records, 632 studies were screened by title and abstract. Subsequently, 602 studies were excluded because they were review articles, meta-analyses, case reports, conference abstracts or basic medical research reports. Then, a comprehensive assessment of the eligibility of 51 full-text articles was conducted, of which 41 studies were excluded for reasons such as lack of complete data and patient overlap between two studies. Finally, 10 studies were included in this meta-analysis, and the detailed characteristics of these included studies are shown in **Tables 1**, **2**. All the studies were conducted mainly in Europe and America, four of which were conducted in the United States (17–19, 24), one in Portugal, one in Spain, one in Israel, one in Poland, two in Norway, and one in Germany. The sample sizes ranged from 30 to 202, and the total number of patients was 929. Nine studies reported patient sex, nine studies reported age, five studies reported AJCC stage, and three studies reported Clark level. Ki-67 values were determined by immunohistochemistry (IHC) in all studies, with cut-off values ranging from 5% to 40%. Of all the studies, eight were retrospective studies, and two were prospective studies. The NOS scores ranged from 6 to 8, with a median value of 7. The follow-up period of the studies ranged from 19.2 months to 151 months.

**Table 1:** Characteristics of the included studies.

Author	Country	Patients		Duratin	Study type	NOS score	ki-67	HR and 95% CI	Survival analysis
Rui et al. (16)	Portugal		82	1990-1996	Retrospective	8	14%	Calculated	OS
Stefan et al. (17)	American&Spain		66	1991-2016	Retrospective	7	10%	Reported	PFS
Ting et al. (18)	American		114	2002-2008	Prospective	7	25%	Reported	OS
Eric M et al. (19)	American		68	2002-2015	Prospective	6	25%	Reported	OS;RFS
O Ben-Izhak et al. (20)	Israel		30	NA	Retrospective	7	40%	Calculated	OS
Oddbjørn et al. (21)	Norway		202	1981-1997	Retrospective	8	16%	Reported	OS
ALEKSANDER et al. (22)	Poland		93	1983-1991	Retrospective	7	20%	Calculated	OS
Vivi Ann et al. (23)	Norway		47	NA	Retrospective	7	5%	Reported	RFS
Nicholas et al. (24)	American		66	1991-2013	Retrospective	7	10%	Reported	PFS
Philipp et al. (25)	Germany		161	1980-2008	Retrospective	7	20%	Reported	OS

CI, confidence interval; HR, hazard ratio; OS, overall survival; PFS, progression-free survival; RFS, relapse-free survival. NA, not available.

**Table 2:** Characteristics of patients enrolled in these studies.

Author	Patients	Gender M/F	Age(years)	AJCC Stage	Anatomic site	Ulceration	Follow-up(months)	Relapse& Metastasis
Rui et al. (16)	82	22/60	NA	I-III	Axial 38; Extremities 46	51/82	37.5 [1-103]	24/82
Stefan et al. (17)	66	42/24	70 [38-95]	NA	Axial 55; Extremities 11	NA	62 [0.5-229]	20/66
Ting et al. (18)	114	63/51	57 [15-92]	I-IV	Axial 57; Extremities 36	32/114	19.2	NA
Eric M et al. (19)	68	40/28	65.4 [26.2-87.4]	II-III	Axial 32; Extremities 36	48/68	31.2	40/68
O Ben-Izhak et al. (20)	30	10/20	16 [1-140]	NA	NA	30/30	16[1-140]	4/30
Oddbjørn et al. (21)	202	90/112	64.4	I-IV	Axial 106; Extremities 95	83/202	76[13-210]	NA
ALEKSANDER et al. (22)	93	35/58	48 [17-78]	I-III	Axial 44; Extremities 49	63/93	44.7[2-116]	50/93
Vivi Ann et al. (23)	47	NA	54 [19-88]	NA	NA	NA	151[26-172]	NA
Nicholas et al. (24)	66	44/22	71 [34-97]	NA	Axial 60; Extremities 6	NA	49[2-268]	37/66
Philipp et al. (25)	161	69/92	55 [22-89]	I-II	NA	NA	122[8-328]	NA

AJCC, American Joint Committee on Cancer; F, female; M, male; NA, not available.

### 3.2 Association Between Ki-67 Expression and OS, PFS and RFS Rates

**Figure 2:**
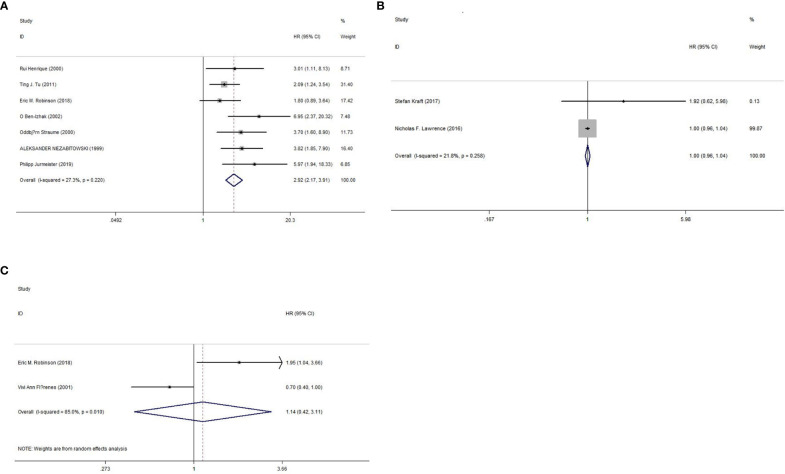
Forest plot of OS, PFS, and RFS. **(A)** Meta-analysis of Ki-67 expression and OS. **(B)** Meta-analysis of Ki-67 expression and PFS. **(C)** Meta-analysis of Ki-67 expression and RFS.

The prognostic value of Ki-67 for the OS rate was reported in seven studies (16, 18–22, 25), and two studies each reported the prognostic value of Ki-67 for PFS (17, 24) and RFS (19, 23) rates. As shown in **Figure 2** and **Table 3**, we used a fixed-effects model because of nonsignificant heterogeneity (I2 = 27.30%, p=0.22), and the results showed that high Ki-67 expression predicted poor OS outcomes (HR=2.92, 95% CI=2.17-3.91, p<0.000). However, there was no significant correlation between high expression of Ki-67 and PFS (HR=0.999, 95% CI =0.958-1.041, P =0.958; I2 = 21.80%, P =0.258) or RFS (HR=1.14, 95% CI = 0.42-3.11, P =0.993; I2 = 85.00%, P =0.01) rates. In addition, we also performed subgroup analysis by region, patients, median age, study type, Ki-67% and follow-up length. As shown in **Table 4**, the OS rate of melanoma patients did not differ between the European, American and Asian populations, between sample sizes greater than or less than 100, between patients with a median age below 50 years and those over 50 years, between retrospective and prospective studies, between Ki-67 values greater than or less than 25%, and between follow-up times greater than or less than 48 months. However, only two studies were included in the PFS and RFS survival analysis, so no subgroup analysis was performed in this study.

**Table 3:** Summary of the meta-analysis of Ki-67 expression and OS, PFS, RFS.

Outcome	Studies	HR	P-value	95% CI	Heterogeneity		
					I2(%)	P-value	Effects model
OS	7	2.92	0.000	[2.17-3.91]	27.30%	0.22	Fixed
PFS	2	0.999	0.958	[0.958-1.041]	21.80%	0.258	Fixed
RFS	2	1.14	0.993	[0.42-3.11]	85.00%	0.01	Random

**Table 4:** Subgroup analysis of pooled HR for melanoma patients with Ki-67 overexpression.

			Overall survival			Heterogeneity		
Group factors	Subgroup	Studies	Pooled HR	P-value	95% CI	I2(%)	P	Effects model
All	All	7	2.92	0.000	[2.17-3.91]	27.30%	0.22	Fixed
region	Europe and America	6	2.72	0.000	[2.00-3.69]	9.80%	0.353	Fixed
	Asia	1	6.95	0.0004	[2.37-20.32]	NA	NA	NA
patients	>=100	3	2.759	0.000	[1.821-4.182]	40.10%	0.188	Fixed
	<100	4	3.084	0.000	[2.035-4.673]	37.20%	0.189	Fixed
median age	>=50	4	2.471	0.000	[1.727-3.535]	31.60%	0.223	Fixed
	<50	2	4.608	0.000	[2.525-8.409]	0.00%	0.366	Fixed
study type	prospective	2	1.982	0.001	[1.301-3.018]	0.00%	0.739	Fixed
	retrospective	5	4.219	0.000	[2.798-6.362]	0.00%	0.773	Fixed
ki-67%	>=25%	3	2.6	0.004	[1.361-4.969]	57.00%	0.1843	Random
	<25%	4	3.873	0.000	[2.483-6.042]	0.00%	0.843	Fixed
follow-up	>=48	2	4.413	0.000	[2.232-8.725]	0.00%	0.508	Fixed
	<48	4	2.408	0.000	[1.711-3.389]	0.00%	0.443	Fixed

NA, not available.

### 3.3 Relationships Between Ki-67 Expression and Clinicopathologic Parameters

We investigated the relationship between the expression of Ki-67 and multiple clinicopathological factors, such as sex, location, ulceration, thickness and vascular invasion. As shown in **Table 5**, Ki-67 overexpression was associated with thickness >4.0 mm (OR=3.09, 95% CI=1.34-7.10, P=0.008; *I* ^2 =^ 0.00%, p=0.351). However, Ki-67 overexpression was not significantly correlated with sex (OR=1.65, 95% CI=0.84-3.25, p=0.149), location (OR=1.43, 95% CI=0.67-3.09, p=0.357), ulceration (OR=5.08, 95% CI=0.73-35.37, p=0.100) or vascular invasion (OR=1.13, 95% CI=0.32-4.00, p=0.855).

**Table 5:** The relationships between Ki-67 expression and clinicopathologic parameters.

Variables	Studies	OR	P-value	95% CI	Heterogeneity		Effects model
					I2(%)	P	
Gender (male *versus* female)	3	1.65	0.149	[0.84-3.25]	19.60%	0.288	Fixed
Location (head and neck *versus* others)	2	1.43	0.357	[0.67-3.09]	4.80%	0.305	Fixed
Ulceration (present *versus* absent)	2	5.08	0.100	[0.73-35.37]	65.10%	0.09	Random
Thickness (mm) (>4.0 *versus* <=4.0)	2	3.09	0.008	[1.34-7.10]	0.00%	0.351	Fixed
Vascular invasion (present *versus* absent)	2	1.13	0.855	[0.32-4.00]	0.00%	0.328	Fixed

### 3.4 Sensitivity Analysis

**Figure 3:**
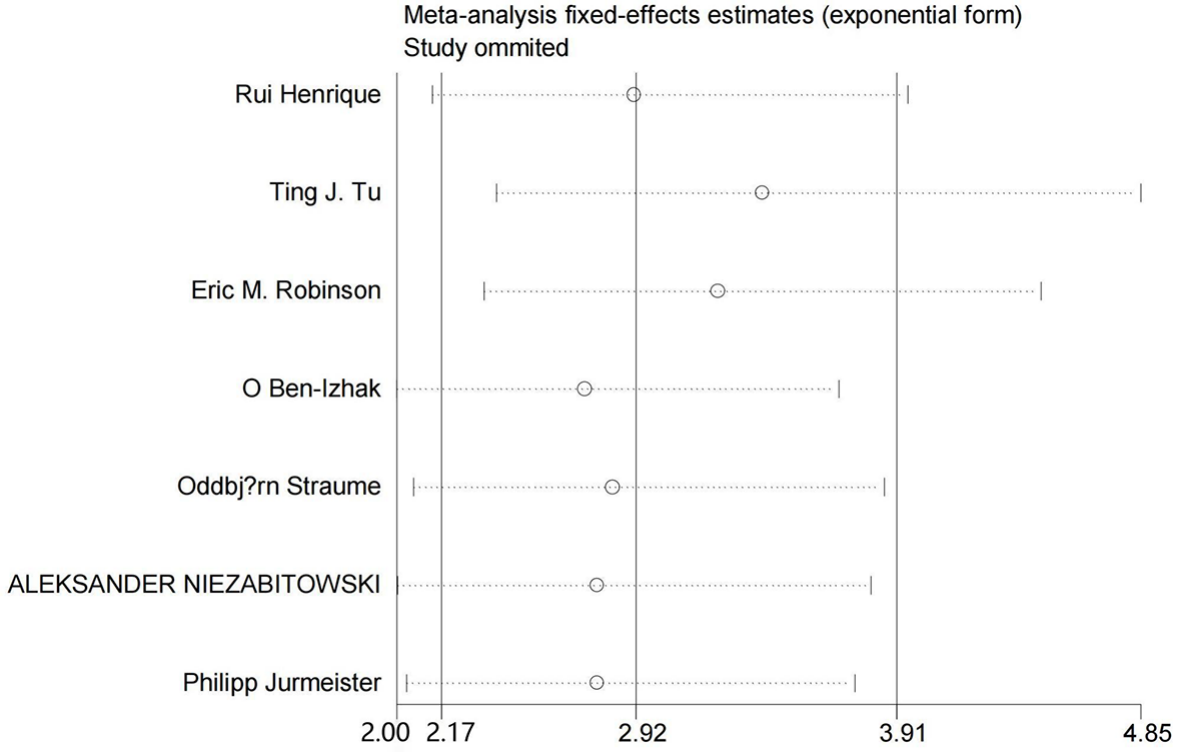
Sensitivity analysis for OS.

To assess the stability of the results, a sensitivity analysis for sequence deletion was performed for each study. We only conducted sensitivity analysis for OS outcomes because only 2 studies reported the PFS and RFS rates. As shown in **Figure 3**, the results showed that no separate study significantly affected the overall HR, which suggested that the results of this meta-analysis are reliable.

### 3.5 Publication Bias

**Figure 4:**
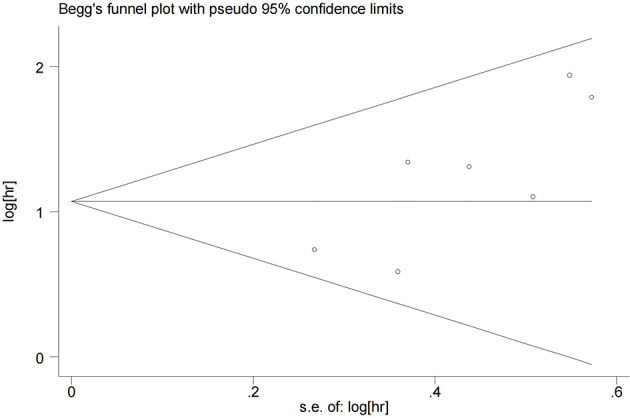
Funnel plots for detecting publication bias of the association between Ki-67 expression level and overall survival of melanoma.

Begg’s test was performed to evaluate publication bias. As shown in **Figure 4**, the funnel plot did not indicate publication bias in the OS outcome (P=0.072). However, both PFS and RFS outcomes were only included in two studies, so it was unnecessary to determine whether there was publication bias in the PFS and RFS analysis.

## 4 Discussion

Currently, the association between ki-67 expression levels and prognosis in melanoma patients is not clear. In this study, we integrated 10 clinical studies to determine the prognostic value of Ki-67 expression in melanoma patients. The results showed that Ki-67 expression was connected with different survival endpoints,including OS, PFS and RFS rate, suggesting that Ki-67 could be used as a valuable index in the prognostication of patients with melanoma. The pooled data also showed that high expression of Ki-67 was associated with melanoma thickness but not with sex, location, ulceration, or vascular invasion. In addition, subgroup analyses indicated that a high level of Ki-67 expression was related to poor OS outcomes in melanoma patients regardless of region, patients, median age of the patients, study type, cut-off of Ki-67% and length of follow-up. Taken together, this is the first study to reveal that high Ki-67 expression is associated with poor prognosis in melanoma patients by using meta-analysis approach.

As the best marker to evaluate cell proliferation, Ki-67 is expressed in all active stages of the cell cycle, including G1, S, G2 and mitosis (28). Therefore, the higher the expression level of KI-67, the faster the tumor growth and the larger the tumor volume will be, leading to the worse prognosis of patients. KI-67 has been proved to be an prognostic index for multiple solid tumors, for example, high ki-67 expression is closely associated with poor OS and DFS (Disease Free Survival) in lung adenocarcinoma (29). A meta-analysis including 8 studies showed that high ki-67 expression was associated with poor OS, PFS, and DMFS (distant metastasis-free survival) in patients with nasopharyngeal carcinoma (8). In hepatocellular carcinoma, high expression of KI-67 has been associated with poor DFS, RFS, and OS (14). Qiu et al. reported that Ki-67 overexpression was correlated with poor OS in patients with ovarian cancer (30). In our study, we revealed that the high expression of KI-67 was closely associated with poor OS in melanoma patients, which is consistent with the conclusion of other solid tumors. However, the reasons why Ki-67 is not related to PFS and RFS may be as follows. First, there are only two studies including PFS and RFS, so there is maybe some bias in the conclusion. Second, high expression of KI-67 may indeed be unrelated to poor PFS and RFS, but the current research data are insufficient, and we expect more data to confirm this conclusion in the future.

Previous studies have shown that the expression level of KI-67 is closely related to the tumor size, such as bladder cancer (31), hepatocellular carcinoma (14), and gastric cancer (32). In our study, we found the relationship between high expression of Ki-67 and melanoma thickness. Thus, the previous findings of the correlations of Ki-67 expression and tomor size in various solid cancers are in accordance with the present study.A recent study showed that melanoma tumor thickness is strongly associated with poor 5-year OS (33). Therefore, the high expression of KI-67 indicates the poor prognosis of melanoma patients, which may be related to tumor thickness. However, a study examining the relationship between ki-67 expression and patient prognosis in thick melanoma (≥4 mm) showed that KI-67 remains an index of poor prognosis in melanoma (19). Therefore, we believe that Ki-67 is still a marker of poor prognosis in melanoma patients even after removing the effect of tumor thickness. Furthermore, ki-67 expression was strongly associated with increased Breslow thickness, Clark level, ulceration, lymphovascular invasion, number of mitosis, and pT stage (34). However, pooled data of this study showed that high ki-67 expression was independent of gender, location, ulceration, or vascular invasion, which may account for the limited sample size of this study.

The advantages of our study are as follows. First, this is the first study to use a meta-analysis to demonstrate that Ki-67 overexpression is a predictor of poor prognosis in melanoma patients. Second, we did a through search to find the best fitting studies, and finally a total of 10 previours studies were included in our meta-analysis. Third, the expression level of KI-67 in the 10 eligible studies included was detected by IHC, which ensured the reliability of the results. Finally, only high quality English literature is included in this study to reduce errors and ensure the authenticity of research conclusions.

However, there are several limitations in our meta-analysis. First, both the number of studies and the total sample size were small. Second, the cut-off point for Ki-67 positivity was different among the included studies, which may have led to heterogeneity. Third, clinical data from Asian and African countries are scarce. Therefore, we need more data from other region groups to determine the influence of region on the study results. Forth, different types of melanomas behave differently, having different molecular signitures and Ki67 proliferation index may have different impacts in those types of melanomas. Finally, as with all meta analysis,it cannot correct some of the bias in the included original studies, and some of the studies which were included in this article were too small for statistical analysis. Therefore, we need more comprehensive designs and large-scale clinical trials for further investigation.

Despite some limitations, our meta-analysis conclusively indicates that Ki-67 overexpression is associated with worse OS rates in melanoma patients. Ki-67 can be used as an important reference marker when evaluating the survival outcomes and prognoses of melanoma patients. Therefore, our study can provide some reference for clinicians in the formulation of melanoma diagnosis and treatment plan, rational allocation of medical resources and preliminary judgment of patient prognosis. There are some shortcomings in our study; thus, we look forward to the completion of more prospective multicentric clinical studies with reasonable designs and on larger scales to verify and supplement our conclusions.

## Data Availability Statement

The original contributions presented in the study are included in the article/supplementary material. Further inquiries can be directed to the corresponding author.

## Author Contributions

QL, ZP, and LSe conceived and designed the study. QL and ZP performed the analysis, prepared the figures and tables and wrote the main manuscript. LFS reviewed and revised the manuscript. All authors contributed to the article and approved the submitted version.

## Conflict of Interest

The authors declare that the research was conducted in the absence of any commercial or financial relationships that could be construed as a potential conflict of interest.

## Publisher’s Note

All claims expressed in this article are solely those of the authors and do not necessarily represent those of their affiliated organizations, or those of the publisher, the editors and the reviewers. Any product that may be evaluated in this article, or claim that may be made by its manufacturer, is not guaranteed or endorsed by the publisher.
